# Long-Term Follow-Up of Peritoneal Interposition Flap in Symptomatic Lymphocele Reduction following Robot-Assisted Radical Prostatectomy: Insights from the PIANOFORTE Trial

**DOI:** 10.3390/cancers16101932

**Published:** 2024-05-19

**Authors:** Christopher Goßler, Matthias May, Steffen Weikert, Sebastian Lenart, Anton Ponholzer, Christina Dreissig, Gjoko Stojanoski, Isabel Anzinger, Josef Riester, Maximilian Burger, Christian Gilfrich, Roman Mayr, Johannes Bründl

**Affiliations:** 1 Department of Urology, Caritas St. Josef Medical Centre, University of Regensburg, 93053 Regensburg, Germany; jriester@csj.de (J.R.); mburger@csj.de (M.B.); roman.mayr@ukr.de (R.M.); johannes.bruendl@ukr.de (J.B.); 2 Department of Urology, St. Elisabeth Hospital Straubing, Brothers of Mercy Hospital, 94315 Straubing, Germany; matthias.may@klinikum-straubing.de (M.M.); christian.gilfrich@klinikum-straubing.de (C.G.); 3 Department of Urology, Alexianer Hospital St. Hedwig, 10115 Berlin, Germany; s.weikert@alexianer.de (S.W.); c.dreissig@alexianer.de (C.D.); 4 Department of Urology and Andrology, St. John of God Hospital Vienna, Brothers of Mercy Hospital, 1020 Vienna, Austria; sebastian.lenart@bbwien.at (S.L.); anton.ponholzer@bbwien.at (A.P.); 5 Department of Urology, Varisano Hospital Frankfurt Höchst, 65929 Frankfurt am Main, Germany; gjoko.stojanoski@varisano.de; 6 Department of Urology, Mallersdorf Hospital, 84066 Mallersdorf, Germany; anzinger.i@klinik-mallersdorf.de

**Keywords:** RARP, prostatic neoplasia, prostate cancer, lymphocele, robotics

## Abstract

**Simple Summary:**

Lymphoceles (lymphatic fluid collections in the pelvis) are a common complication of robot-assisted radical prostatectomy. Peritoneal interposition flaps have been proposed as an intraoperative modification to reduce lymphocele formation. However, data from randomised controlled trials on this subject are not conclusive. In particular, data on long-term efficacy and complications are lacking. The PIANOFORTE trial was the first randomised controlled trial exploring this subject and showed a negative outcome regarding lymphocele reduction by performing a peritoneal interposition flap. In this long-term follow-up of the trial (median 43 months postoperatively), we could confirm the initial result that, while the interposition flap does not have a negative impact on complications or functionality, there is also no effect on lymphocele reduction in the long term. Additionally, these results confirm the possibility of lymphocele formation beyond the third postoperative month, which has to be borne in mind in the follow-up of these patients.

**Abstract:**

The available randomised controlled trials (RCTs) assessing the influence of peritoneal interposition flaps (PIF) on the reduction of symptomatic lymphoceles (sLCs) post robot-assisted radical prostatectomy (RARP) do not constitute a sufficient follow-up (FU) to assess the long-term effects. The PIANOFORTE trial was the first of these RCTs, showing no sLC reduction at the 3-month FU. Therefore, all 232 patients from the PIANOFORTE trial were invited for long-term FU. One hundred seventy-six patients (76%) presented themselves for FU and constituted the study group (SG). The median FU duration was 43 months. No significant differences in group allocation or LC endpoints at 90 days were observed between SG patients and patients not presenting themselves for the FU. During the FU period, four patients (2.3%) in the SG developed sLCs, and six patients (3.4%) developed asymptomatic lymphoceles (aLCs), which persisted in five patients (2.9%). There were no significant differences between PIF and non-PIF regarding sLC/aLC formation or persistence, newly developed complications, stress urinary incontinence or biochemical/clinical tumour recurrence. Therefore, this long-term FU confirms the primary outcomes of the PIANOFORTE trial that, while PIF does not impact complications or functionality, it does not reduce sLC/aLC rates. Furthermore, it shows the potential occurrence of LC after the third postoperative month.

## 1. Introduction

With 1,414,259 new cases diagnosed worldwide in 2020, prostate cancer (PCa) remains the second most common cancer in men [1]. In the case of organ-confined PCa, robot-assisted radical prostatectomy (RARP) represents the most frequent surgical approach for radical prostatectomy in industrialised nations, as it has shown good oncological and functional results while reducing perioperative morbidity [2,3].

As it provides the most accurate assessment of lymph node staging and prognosis [4], intraoperative pelvic lymph node dissection (PLND) is recommended in high-risk patients as well as intermediate-risk patients with an elevated risk of lymph node involvement [5]. However, PNLD remains a relevant cause of postoperative complications, most notably lymphocele (LC) formation [4].

Symptomatic lymphoceles (sLCs) have been reported in up to 2–10% of patients after RARP [6,7,8]. Various alterations of the intraoperative technique have been evaluated, with the aim of reducing postoperative sLCs, including the peritoneal interposition flap (PIF) [9].

At present, there are outcomes from six randomised-controlled trials (RCTs) that examined the impact of a PIF on the rate of sLCs and asymptomatic lymphoceles (aLCs) during follow-ups ranging from 90 days (Pianoforte, ProLy) to 595 days (PerFix). The median follow-up duration across all six studies was 145 days (interquartile range (IQR) 90–365 days) [10]. An aggregated analysis of all six RCTs currently available, comprising a cohort of 2792 patients, revealed an sLC incidence of 5.0% (69/1381) in the intervention group compared to 8.4% (119/1411) in the control group (OR: 0.53; 95%-CI: 0.34–0.84) [10]. However, three out of the six included RCTs showed a negative result, meaning there was no significant reduction in the sLC rate by the use of PIF. Consequently, based on the current dataset, it remains unclear what influence PIF has beyond the first and second postoperative years on sLC and aLC rates, as well as which late-time impacts of other nature might manifest (complications independent of LCs, stress urinary incontinence (SUI), etc.).

The PIANOFORTE trial [11] was the first of these RCTs and also showed no significant reduction in sLC and aLC rates by performing PIF. We have now conducted the first extended follow-up of an RCT investigating the implications of PIF on LC rates, complications, functionality and oncologic outcome in the long term (with a minimum of 3 years of follow-up).

## 2. Materials and Methods

### 2.1. Study Population and Follow-Up

**Figure 1:**
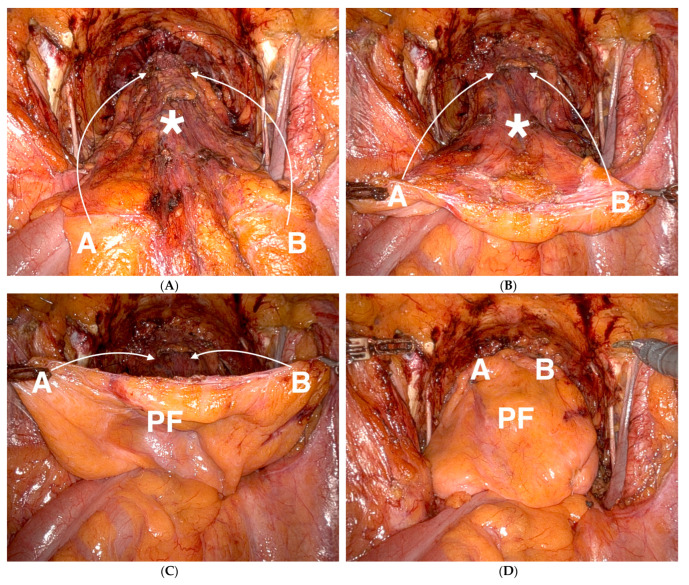
Peritoneal interposition flap (intraoperative view of the pelvis). (**A**–**C**): After completion of the vesicourethral anastomosis (*) and bilateral pelvic lymph node dissection (PLND), the cranial margin (points A and B) of the future peritoneal interposition flap (PF) is fixed to the perivesical adipose tissue by clips. (**D**): After completion of the PIF, the anterior and lateral aspects of the bladder (facing the PLND bed) are covered with peritoneum.

The study cohort consisted of 232 patients with clinically organ-confined PCa and cM0-status who underwent transperitoneal RARP with simultaneous bilateral PLND between March 2017 and December 2017 in one Austrian centre and three German centres within the PIANOFORTE trial (impact of peritoneal flap on outcome after robotic prostatectomy) [11]. The trial had previously been approved by the appropriate institutional ethics committees and was registered in the clinical trials registry (DRKS-ID: DRKS00011115) [12]. The trial’s inclusion and exclusion criteria, patient characteristics and detailed surgical technique have been described in the original publication [11]. Per study protocol, all patients had a follow-up performed (either in their study centre or as outpatients) 90 days postoperatively. PIF, if applicable according to patients’ randomisation, was performed as described in the original publication [11]. A visual depiction of intraoperative PIF can be found in Figure 1.

For the current long-term follow-up, all patients were invited to another examination at their respective study centres. As per the original study protocol, the primary endpoint of this long-term follow-up was the sLC rate at the time of follow-up. Secondary endpoints were LC volume at the time of follow-up, LC treatment that may have been performed as well as any other complications that may have occurred between the 90 days and the long-term follow-up examination (graded according to the Clavien–Dindo classification system [13]), as well as current SUI at the time of follow-up (classified in severity according to the Ingelman–Sundberg scale [14,15,16]). Therefore, when patients presented for examination, an abdominal ultrasound (US) was conducted to scan for pelvic LCs. Furthermore, patients were asked to complete a questionnaire to assess whether or not sLCs had been detected and treated between the 90 days and the long-term follow-up examination, current SUI, any other complications that may have occurred between the 90 days and the long-term follow-up examination, as well as the current oncologic status, including prostate-specific antigen (PSA).

As per the original study protocol, an asymptomatic LC was defined as a pelvic collection of fluid solely detected by US. sLCs, on the other hand, were defined as LCs accompanied by either rheological complications (thromboembolic complications, lymphedema), LC infection, lower urinary tract symptoms or abdominal pain in the region of the LC.

### 2.2. Statistical Analysis

Categorical endpoints were reported as absolute and relative frequencies, continuous variables as median and interquartile range (IQR). The Mann–Whitney U-test was used to differentiate the distribution of continuous variables between the two groups (PIF vs. no PIF). The distribution of categorical variables was analysed using the Chi-squared test (in case of 2 × 2 contingency tables: Fisher’s exact test).

To reduce a possible selection bias regarding the patients presenting for long-term FU, a baseline comparison between patients who presented for long-term FU and those lost to long-term FU was performed. This included the patients’ group in the original PIANOFORTE trial (PIF vs. no PIF), their age as well as the endpoints of the original trial.

Data analysis was carried out using IBM SPSS Statistics for Windows, version 29.0 (IBM Corp., Armonk, NY, USA). All mentioned *p*-values are two-tailed; the significance level was defined as *p* < 0.05.

## 3. Results

Of the original 232 patients included in the PIANOFORTE trial, a follow-up could be obtained for 176 patients (75.9%) who were considered the study group (SG). There were 4 patients (1.7%) who had previously died and 52 patients (22.4%) who were lost to follow-up. Median follow-up was 43 months (interquartile range (IQR) 41.0–46.0 months) and did not differ between the two groups. A detailed overview of the patients presenting for long-term follow-up can be found in Table 1. To ensure comparability between the two groups (PIF vs. no PIF) regarding functional and oncological follow-up, this analysis also included the oncological outcome at RARP (tumour stage, International Society for Urological Pathology (ISUP) grading, lymph node status, positive surgical margins) as well as previous abdominal surgery prior to RARP.

To exclude a possible selection bias, patients who presented for long-term follow-up and those lost to long-term follow-up were compared with regard to their age, their group in the original PIANOFORTE trial (PIF vs. no PIF) as well as LC formation at 90 days after surgery. In this analysis, there was a significant difference regarding age at the time of surgery between both groups, with patients presenting for long-term follow-up being a median of four years younger than those lost to long-term follow-up (median age (IQR) 64.5 years (58.25–68.0) vs. 68.5 years (62.0–73.0), *p* = 0.003). However, no significant differences between both groups could be found regarding the original study endpoints at 90 days post-surgery (Supplementary Table S1). Therefore, the present cohort of patients presenting for long-term follow-up can be considered as a representative cross-section of the original study population.

During the follow-up period, four patients (2.3%) in the SG developed sLCs, and six patients (3.4%) in the SG developed aLCs (in one case, this could be attributed to a radio-guided salvage LND between 90 days and long-term follow-up). One of these patients had required LC therapy (at long-term follow-up, no LC was found); in four patients, LC had spontaneously resolved; and in five of those patients (2.8% of the SG), aLCs were persistent at the time of long-term follow-up (in four cases, the LC were less than 30 mL; in the remaining patient, it was less than 100 mL). The other 171 patients of the SG (97.2%) did not show any remaining LC. When comparing the SG patients regarding PIF vs. non-PIF, no significant differences could be found in sLC (2.5% vs. 2.1%) or aLC (4.9% vs. 2.1%) formation (*p* = 0.576) or persistence (4.9% vs. 1.1%, *p* = 0.274).

There were also no statistically significant differences between the PIF and non-PIF group regarding stress urinary incontinence (*p* = 0.981) or other newly developed complications (*p* = 0.481), especially when considering complications of Clavien–Dindo grade ≥3a (*p* = 0.999). One patient (0.6%) had experienced a secondary insufficiency of the vesico-urethral anastomosis, four patients (2.3%) had experienced thromboembolic complications, and 16 patients (9.1%) reported other complications (hernia in four patients (2.3%), stricture of the vesico-urethral anastomosis in five patients (2.8%), suprapubic pain and gross haematuria in three patients (1.7%), febrile urinary tract infection (UTI) in three patients (1.7%) and asymptomatic hydrocele in one patient (0.6%)).

An oncologic outcome could be assessed in 175 of the 176 patients reporting for long-term follow-up (one patient had decided against PSA-based follow-up). At their last oncological follow-up, 76.7% of the SG (135 of 176 patients) had shown no evidence of biochemical or clinical tumour recurrence, with no statistically significant intergroup disparity (*p* = 0.287).

## 4. Discussion

Although it is a relevant comorbidity factor and its curative potential is still unclear [4,6], PLND remains an essential part of the procedure in RARP for patients with high-risk or intermediate-risk localised PCa [5] due to its role in the staging of metastatic lymph node involvement [4]. As LC formation is the most frequent complication of PLND [4], several modifications of the surgical technique have been analysed regarding their effect on postoperative LC formation, most notably the PIF [9].

Recently, a systematic review comprising four RCTs (PIANOFORTE, PerFix, ProLy and PLUS) reported a significant risk reduction regarding sLC formation by performing intraoperative PIF [17]. Another recent meta-analysis included a fifth RCT, the PELYCAN trial, and found a reduction in postoperative LC formation but not in sLC formation [18]. Finally, the most recent systematic review on this matter [10] included a sixth RCT [19,20,21,22,23]. As previously mentioned, the median follow-up duration across all six studies was 145 days (interquartile range (IQR) 90–365 days) [10]. A detailed presentation of all six RCTs on the impact of PIF on sLC reduction can be found in Table 2. The ProLy trial [19], the PerFix trial [20] and the PELYCAN trial [22] showed a significant reduction in LC and sLC formation due to the PIF, while the PIANOFORTE trial [11], the PLUS trial [21] and Pose et al. [23] did not.

In our long-term follow-up of the PIANOFORTE trial, the median follow-up was 43 months after RARP (IQR: 41–46 months), with a minimum of exactly 36 months, which, to the best of our knowledge, so far has not been reached by other trials [18]. Further trials are continuously performed, with only short-term results available at this time, however [24].

At the time of our follow-up, we found five patients with aLCs, while no sLCs could be found. However, when examining the patients’ histories by means of the filled-out questionnaires, a history of LC formation could be found in five more patients, whose LC had in one case (sLC) been adequately treated and in the other four cases (three of them sLCs) had spontaneously resolved in the meantime. In one aLC patient, radio-guided salvage LND was performed between RARP and follow-up, and it was found to be responsible for the aLC formation, as no LC had been diagnosed 90 days postoperatively. As there were no statistically significant differences between both groups (PIF or no PIF) regarding either LC at follow-up or LC in the patients’ history as well as previous abdominal surgery prior to RARP (therefore excluding a possible confounding effect), this confirms this trial’s initial results from the 90 days follow-up that there is no reduction in LC formation by means of PIF during RARP.

In the original publication, at 90 days after RARP, a total of 49 patients presented with LCs. Of these patients, 41 were available for follow-up, and four patients were among those with persistent LCs at long-term follow-up. One patient had received therapy shortly after the 90-day follow-up, which means that in at least 36 of 49 patients (75.5%), LCs had spontaneously resolved between 90 days postoperatively and long-term follow-up. This confirms the current literature that most LCs resolve spontaneously [25].

It has been shown that the risk of LC formation after RARP increases with the extent of PLND and, therefore, the median lymph node yield, reaching a plateau at about 13 removed lymph nodes [26,27,28]. Thus, it could be discussed that performing PIF might be reasonable in patients receiving extended PLND. However, the median lymph node yield in all six previously cited RCTs was at least 14 lymph nodes in extended PLND. Therefore, a higher efficacy of PIF in patients receiving extended PLND cannot be supported.

Venous thromboembolism is a well-documented and serious complication of pelvic LC formation [29]. However, none of the four patients presenting at long-term follow-up with a history of thromboembolism had an LC either at 90 days postoperatively, at long-term follow-up or in their history in between. This underlines the need for thromboembolism prophylaxis in all RARP patients.

With regard to the oncological outcomes, which were not a primary endpoint of this long-term follow-up, Novara et al. reported in their systematic review and meta-analysis seven-year BCR-free survival estimates of approximately 80% [30]. In our study cohort, a similar BCR-free survival rate of 77.1% was found after a median follow-up of 43 months. This might correlate to the initial rate of patients with negative surgical margins after RARP, which had been 86.4% with no significant inter-group disparity. Due to the small sample size, however, we refrain from drawing any definitive conclusions.

Modification of the RARP technique with PIF is generally considered a safe procedure [17,18]. With regard to other complications, such as chronic suprapubic pain or hernia, there were no statistically significant differences between the original study group and the control group at long-term follow-up. These complications were systematically assessed according to the Clavien–Dindo classification [13], and no significant differences regarding the rates of minor complications (Clavien–Dindo ≤ 2) or complications requiring intervention (Clavien–Dindo ≥ 3a) were found. Therefore, we agree that PIF is a feasible procedure that does not cause patients to have a higher risk of impairment in the long term.

Our study is not free of limitations. For one, the long-term follow-up has been achieved in only 75.9% of the original study cohort. Secondly, events prior to the long-term follow-up (LC therapy, other complications) were assessed by means of questionnaires completed by the patients themselves. Thirdly, the originally deployed double-blind technique (meaning neither the patient nor the outpatient urologist conducting the 90-day follow-up was informed whether the patient had received PIF or not) could not be employed in the long-term follow-up analysis. Finally, an LC assessment was conducted by ultrasound by multiple different investigators.

## 5. Conclusions

In this first long-term follow-up of a randomised controlled trial regarding the impact of PIF on LC formation, we found that there was no significant difference in LC formation whether PIF was performed or not. However, there were also no significant differences with regard to long-term complications. Based on these long-term findings, as well as the mixed results from the existing RCTs, a clear recommendation for performing PIF cannot be given at this time, even with regard to extended PLND. As the study group in this follow-up was already rather small, another follow-up in the future would be unlikely to yield sufficiently valid results. Additional long-term data of the three positive RCTs would be necessary to shed further light on the efficacy of PIF in sLC reduction after RARP.

## Figures and Tables

**Table 1:** Distribution of study criteria at long-term follow-up depending on intervention (PIF vs. no PIF).

Criteria	Whole Study Group	PIF	No PIF	*p*
Follow-up in months, median (IQR)	43.0 (41.0–46.0)	42.0 (40.0–46.0)	43.0 (41.0–45.00)	0.758
Patients presenting for long-term FU (% of the original study population)	176 (75.9%)	81 (75.0%)	95 (76.6%)	0.516
**All further values and percentages refer to the above-mentioned number of patients** **presenting for long-term FU**				
Patients’ age at the time of RARP in years, median (IQR)	64.5 (58.25–68.0)	64.0 (58.5–67.5)	65.0 (58.0–70.0)	0.454
ISUP grading score in RARP				0.559
1	12 (6.8%)	7 (8.6%)	5 (5.3%)	
2	94 (53.4%)	40 (49.4%)	54 (56.8%)	
3	42 (23.9%)	23 (28.4%)	19 (20.0%	
4	18 (10.2%)	7 (8.6%)	11 (11.6%)	
5	10 (5.7%)	4 (4.9%)	6 (6.3%)	
T-Stage in RARP				0.111
pT0	1 (0.6%)	1 (1.2%)	0 (0%)	
pT2a	15 (8.5%)	10 (12.3%)	5 (5.3%)	
pT2b	11 (6.3%)	6 (7.4%)	5 (5.3%)	
pT2c	100 (56.8%)	37 (45.7%)	63 (66.3%)	
pT3a	26 (14.8%)	14 (17.3%)	12 (12.6%)	
pT3b	22 (12.5%)	13 (16.0%)	9 (9.5%)	
pT4	1 (0.6%)	0 (0%)	1 (1.1%)	
Positive lymph nodes in RARP				0.550
No	164 (93.2%)	77 (95.1%)	87 (91.6%)	
Yes	12 (6.8%)	4 (4.9%)	8 (8.4%)	
Positive surgical margins in RARP				0.509
No	152 (86.4%)	68 (84.0%)	84 (88.4%)	
Yes	24 (13.6%)	13 (16.0%)	11 (11.6%)	
Abdominal surgery prior to RARP				0.622
None	96 (54.5%)	44 (54.3%)	52 (54.7%)	
Minor	68 (38.6%)	33 (40.7%)	35 (36.8%)	
Major (laparotomy)	12 (6.8%)	4 (4.9%)	8 (8.4%)	
LC at long-term FU				0.274
- No LC	171 (97.2%)	77 (95.1%)	94 (98.9%)	
- 0–30 mL	4 (2.3%)	3 (3.7%)	1 (1.1%)	
- 30–100 mL	1 (0.6%)	1 (1.2%)	0 (0.0%)	
Symptoms at long-term FU				0.626
- No symptoms	172 (97.7%)	80 (98.8%)	92 (96.8%)	
- Abdominal pain	4 (1.7%)	1 (1.2%)	3 (3.2%)	
LC between 90 days and long-term FU				0.576
- No LC	166 (94.3%)	75 (92.6%)	92 (95.8%)	
- Asymptomatic	6 (3.4%)	4 (4.9%)	2 (2.1%)	
- Symptomatic	4 (2.3%)	2 (2.5%)	2 (2.1%)	
LC therapy between 90 days and long-term FU				0.999
- No	175 (99.4%)	81 (100.0%)	94 (98.9%)	
- Yes	1 (0.6%)	0 (0.0%)	1 (1.1%)	
- SUI at long-term FU				0.981
- No SUI	113 (64.2%)	51 (63.0%)	62 (65.3%)	
- Grade 1	54 (30.7%)	26 (32.1%)	28 (29.5%)	
- Grade 2	7 ( 4.0%)	3 (3.7%)	4 (4.2%)	
- Grade 3	2 (1.1%)	1 (1.2%)	1 (1.1%)	
Other complications between 90 days and long-term FU (multiple possible)				0.481
- No complications	155 (88.1%)	74 (91.4%)	81 (85.3%)	
- Secondary insufficiency of the vesicourethral anastomosis	1 (0.6%)	0 (0.0%)	1 (1.1%)	
- Thromboembolism	4 (2.3%)	2 (2.5%)	2 (2.1%)	
- Other complications	16 (9.1%)	5 (6.2%)	11 (11.6%)	
Clavien–Dindo classification of above-mentioned complications:				0.999
- Clavien–Dindo ≤ 2	14 (7.9%)	4 (4.9%)	10 (10.6%)	
- Clavien–Dindo ≥ 3a	6 (3.4%)	3 (3.7%)	3 (3.2%)	
Oncologic follow-up				0.287
- No recurrence	135 (76.7%)	58 (71.6%)	77 (81.1%)	
- Biochemical recurrence	21 (11.9%)	13 (16.0%)	8 (8.4%)	
- Confirmed local recurrence	1 (0.6%)	0 (0.0%)	1 (1.1%)	
- Confirmed metastatic disease	18 (10.2%)	10 (12.3%)	8 (8.4%)	
- No oncologic follow-up	1 (0.6%)	0 (0.0%)	1 (1.1%)	

Legend: FU, follow-up; IQR, interquartile range; ISUP, International Society of Urological Pathology; LC, lymphocele; PIF, peritoneal interposition flap; RARP, robot-assisted radical prostatectomy; SUI, stress urinary incontinence.

**Table 2:** Presentation of results from all randomised controlled trials (RCTs) investigating the impact of peritoneal interposition flaps (PIF) on the reduction of symptomatic lymphoceles (sLC) following robot-assisted radical prostatectomy inclusive pelvic lymph node dissection while considering individual follow-up (FU) durations.

Reference (Study Name)	Country	Study Period	Study Design	FU (Months) ^a^	sLC Rate ^d^ *p*-Value	FU Extension (Months)	sLC Rate ^e^ *p*-Value
Bründl 2020 (PIANOFORTE) [11]	Germany, Austria	2017	Multi-centre double-blinded RCT	3 ^b^	Intervention: 9/108 (8.3%) Control: 12/124 (9.7%) *p* = 0.721	43 ^c^	Intervention: 2/81 (2.5%) Control: 2/95 (2.1%) *p* = 0.871
Gloger 2022 (ProLy) [19]	Germany	2018–2020	Multi-centre double-blinded RCT	3 ^b^	Intervention: 8/239 (3.3%) Control: 19/236 (8.1%) *p* = 0.027	n.a.	n.a.
Student 2023 (PerFix) [20]	Czech Republic	2019–2021	Single-center single-blinded RCT	20 ^c^	Intervention: 3/123 (2.4%) Control: 14/122 (11.5%) *p* = 0.005	n.a.	n.a.
Wagner 2023 (PLUS) [21]	United States of America	2018–2021	Single-center assessor-blinded RCT	3.7 ^c^	Intervention: 1/110 (0.9%) Control: 1/106 (0.9%) *p* = 0.979	n.a.	n.a.
Neuberger 2023 (PELYCAN) [22]	Germany	2019–2021	Single-center double-blinded RCT	6 ^b^	Intervention: 10/270 (3.7%) Control: 25/274 (9.1%) *p* = 0.010	n.a.	n.a.
Pose 2023 (Michl-Technique) [23]	Germany	2017–2019	Single-center single-blinded RCT	12 ^b^	Intervention: 38/531 (7.2%) Control: 48/549 (8.7%) *p* = 0.336	n.a.	n.a.

Legend: ^a^ Median FU of all six RCTs: 4.85 (3–12) months. ^b^ FU of each study participant. ^c^ Median FU of the RCT. ^d^ Aggregate rate of sLCs across all six RCTs based on the original publications: 5.0% (69/1381) in the intervention group compared to 8.4% (119/1411) in the control group (*p* < 0.001). ^e^ Rate of newly occurring symptomatic lymphoceles beyond the follow-up period specified in the original publication of the RCT. n.a.: not applicable.

## Data Availability

The data presented in this study are available upon request from the corresponding author due to data protection reasons. The authors confirm that the images and tables from this paper have not been reprinted and have not been previously published.

## Supplementary Materials

The following supporting information can be downloaded at https://www.mdpi.com/article/10.3390/cancers16101932/s1. Table S1: Comparison of patients with long-term follow-up complete and patients lost to long-term follow-up.
