# Supplementary material for: Long-Term Follow-Up of Peritoneal Interposition Flap in Symptomatic Lymphocele Reduction following Robot-Assisted Radical Prostatectomy: Insights from the PIANOFORTE Trial
Source: Cancers (Basel). 2024 May 19;16(10):1932. doi: 10.3390/cancers16101932 (PMC11119833; doi:10.3390/cancers16101932)
Supplement: Supplementary file 1 [file cancers-16-01932-s001.zip › cancers-2987403-supplementary.pdf]

Supplement Tabel S1 Comparison of patients with long-term follow-up complete and patients lost to long-term follow-up

**Criteria**	**Whole study group**	**Lost to long-term FU**	**Long-term FU complete**	**p**
Number of patients	232	56	176	
Patients' age at the time of surgery in years, median (IQR)	65.0 (60.0-70.0)	68.5 (62.0-73.0)	64.5 (58.25-68.0)	**.003**
**All further percentages refer to the above mentioned number of patients with or without long-term follow-up**				
PIF performed, n (%)				.878
- Yes	108 (46.6%)	27 (48.2%)	81 (46.0%)	
- No	124 (53.4%)	29 (51.8%)	95 (54.0%)	
LC volume at time of discharge, n (%)				.982
- No LC	195 (84.1%)	47 (83.9%)	148 (84.1%)	
- <30 ml	24 (10.3%)	5 (8.9%)	19 (10.8%)	
- 30-100 ml	12 (5,2%)	4 (7.1%)	8 (4.6%)	
- 101-200 ml	1 (0.4%)	0 (0%)	1 (0.6%)	
- >200 ml	0 (0%)	0 (0%)	0 (0%)	
Symptomatic LC during inpatient stay, n (%)				.565
- Yes	3 (1.3%)	1 (1.8%)	2 (1.1%)	
- No	229 (98.7%)	55 (98.2%)	174 (98.9%)	
LC therapy during inpatient stay, n (%)				.591
- No LC or no therapy	229 (98.7%)	55 (98.2%)	174 (98.9%)	
- Drainage with sclerotherapy	2 (0.9%)	1 (1.8%)	1 (1.8%)	
- Surgical therapy	1 (0.4%)	0 (0%)	1 (1.8%)	
LC volume at follow-up 90 days after surgery, n (%)				.319
- No LC	183 (78.9%)	48 (85.7%)	135 (76.7%)	
- <30 ml	19 (8.2%)	2 (3.6%)	17 (9.7%)	
- 30-100 ml	17 (7.3%)	1 (1.8%)	16 (9.1%)	
- 101-200 ml	8 (3.4%)	2 (3.6%)	6 (3.4%)	
- 201-300 ml	2 (0.9%)	2 (3.6%)	0 (0%)	
- >300 ml	3 (1.3%)	1 (1.8%)	2 (1.1%)	
Symptomatic LC at follow-up 90 days after surgery, n (%)				.971
- Yes	21 (9.1%)	5 (8.9%)	16 (9.1%)	

- No	211 (90,9%)	51 (91.1%)	160 (90.9%)	
LC therapy in the first 90 days after surgery, n (%)				.760
- No LC or no therapy	211 (90.9%)	51 (91.1%)	160 (90.9%)	
- Drainage without sclerotherapy	2 (0.9%)	1 (1.8%)	1 (0.6%)	
- Drainage with sclerotherapy	12 (5.2%)	2 (3.6%)	10 (5.7%)	
- Surgical therapy	7 (3.0%)	2 (3.6%)	5 (2.8%)	
Complications during the inpatient stay according to the Clavien-Dindo classification, n (%)				.560
- No complications	192 (82.8%)	45 (80.4%)	147 (83.5%)	
- 1	15 (6.5%)	7 (12.5%)	8 (4.6%)	
- 2	6 (2.6%)	1 (1.8%)	5 (2.8%)	
- 3a	10 (4.3%)	1 (1.8%)	9 (5.1%)	
- 3b	9 (3.8%)	2 (3.6%)	7 (4.0%)	
- 4a	0 (%)	0 (%)	0 (%)	
- 4b	0 (%)	0 (%)	0 (%)	
- 5	0 (%)	0 (%)	0 (%)	

Legend: FU, follow-up; LC, lymphocele; PIF, peritoneal interposition flap
